# Reply to Lemans et al. Comment on “Burgos et al. Fusionless All-Pedicle Screws for Posterior Deformity Correction in AIS Immature Patients Permit the Restoration of Normal Vertebral Morphology and Removal of the Instrumentation Once Bone Maturity Is Reached. *J. Clin. Med.* 2023, *12*, 2408”

**DOI:** 10.3390/jcm12144773

**Published:** 2023-07-19

**Authors:** Jesús Burgos, Gonzalo Mariscal, Luis Miguel Antón-Rodrigálvarez, Ignacio Sanpera, Eduardo Hevia, Vicente García, Carlos Barrios

**Affiliations:** 1 Spine Unit, Hospital Viamed Fuensanta, 28027 Madrid, Spain; 2 School of Doctorate, Valencia Catholic University, 46001 Valencia, Spain; gonzalo.mariscal@mail.ucv.es; 3 Pediatric Orthopedics, Ramon y Cajal Hospital, 28034 Madrid, Spain; lmantonrodrigalvarez@gmail.com; 4 Pediatric Orthopedics, Hospital Son Espases, 07198 Palma de Mallorca, Spain; isanpera@gmail.com; 5 Spine Unit, Hopsital La Fraternidad-Muprespa, 28036 Madrid, Spain; eduardoheviasierra@gmail.com; 6 Sección de Cirugía de Columna, Hospital Universitario Araba, 01009 Vitoria, Spain; vigarto@hotmail.com; 7 Institute for Research on Musculoskeletal Disorders, Valencia Catholic University, 46001 Valencia, Spain; carlos.barrios@ucv.es

We thank Dr. Lemans and coworkers for their interest and knowledgeable comments [1] on our study [2].

One of the concerns was the method used to assess the growth modulation. Of course, the only method to evaluate true vertebral growth is through a CT scan. For obvious ethical reasons, patients cannot be subjected to repeated CT scans, which is unnecessary for monitoring AIS patients. In the hypothetical case that it could be performed, artifacts induced by implants would also make it difficult to assess the remodeling of the vertebral bodies. In our study, vertebral wedging could not be assessed postoperatively because of rod overlap; it could only be evaluated once the implants were removed.

Evidently, 2D measurements have certain limitations. Since our measurement is the ratio of vertebral body heights between concavity and convexity, vertebral tilt has very little influence. Spinal rotation apparently has more influence, but the authors know that the wedging of the vertebrae at the apex cannot be simply explained by rotation alone. Interestingly, if the area with the greatest wedging in the concavity rotates toward the convexity, as is common in idiopathic scoliotic deformities, a measurement of the vertebral body height on the concave side would evaluate a more posterolateral area of the vertebral body, that is, with less wedging, and the ratio would be higher than that on the convex side. The true vertebral wedging was greater than that quantified by our wedging ratio. In other words, our measurement in the coronal plane minimized true vertebral wedging. At the end of follow-up, the measurement of the wedging ratio in the coronal plane after implant removal and without vertebral rotation clearly showed almost complete restoration of the vertebral shape; in other words, can the wedging ratio be considered an indirect measure of true wedging? It is an indirect measure, but it undoubtedly permits the assessment of vertebral morphology restoration.

Concerning the risk of curve progression after removal of the instrumentation, we want to point out that in our cases, there is a very low risk of secondary curve progression since the removal of instrumentation is performed at the end of growth; when the vertebral shape is normalized, and wedging is not present, the progression of the curves is minimized. In our cases, the normal anatomy of the vertebral bodies was restored, and therefore, no residual wedging was apparent. In scoliosis surgery, curve progression only occurs if residual vertebral wedging is present, or if a poor curve correction was not accurate after surgery. The problem of incomplete or insufficient correction exists very often after time of surgery. It is unusual to obtain a correction of more than 80% after scoliosis surgery, but it is considered a success. For avoiding fusion using our technique, a correction of more than 80% is an essential requirement. If vertebral bodies acquire an almost normal anatomy without wedging and an almost complete curve, the risk of curve progression is improbable. Our cases have follow-ups more than 2 years after the removal of the instrumentation. During that follow-up period, the measurements of both the coronal and sagittal planes disclosed no more than 2 Cobb degrees, which is considered no progression at all. We think that a follow-up 2 years after implant removal is enough to prove that no more progression of the curves could be expected.

We would appreciate it if the authors of the letter could review the data presented in the quoted articles in more detail. According to the literature provided, there is a significant risk of curve progression when implants are removed, particularly in immature patients in which the restoration of the vertebral shape could not be completed. In our opinion, based on previous experience, the presence of residual wedging justifies the curve progression. Reference #5 included a group of patients in whom implant removal due to complications was performed while they were still skeletally immature with the potential for continued growth, resulting in loss of correction because vertebral wedging was still present. In addition, the authors of the study mentioned that there was also a significant difference in the progression of the curve in the group that underwent instrumentation removal <2 years after surgery compared with >2 years. The latter cases would have a more advanced degree of skeletal maturity, less wedging, and therefore, a lower risk for progression. The same finding was reported in reference article #7. Many of the patients were still skeletally immature at the time of implant removal, meaning that they had no completed vertebral remodeling and therefore had a high probability of loss of correction due to residual wedging. Our experience coincides with those of these authors. We also observed that removal of instrumentation before the completion of growth with complete closure of the iliac physis (Risser 5) can result in progression of the deformity, even in cases with irrelevant residual curves and in those close to complete physeal closure. From the literature, it is reasonable to assume that scoliosis progression in immature patients occurs due to the presence of asymmetric pressures on the vertebral epiphyseal plates, leading to vertebral growth asymmetry that increases the deformity.

With regard to the development of degenerative disc changes in early onset scoliosis, these changes are likely to be multifactorial. Important factors for the development of these changes include the repetitive application of large forces on intervertebral discs in non-physiological positions, on discs that are not perpendicular to gravitational loads, and prolonged periods of vertebral immobilization to which these patients are subjected. This situation is not comparable to that in our methodology, in which complete correction of the deformity is essential. In our cases, no new asymmetrical overloads were applied to these elements during the immobilization period. In our technique, on the concave side, the pressure on the disc is reduced by the instrumentation (distraction forces) allowing epiphyseal growth, while at the convexity, compression forces block the growth on that side. The asymmetric forces were progressively balanced.

Certainly, there is a possibility of developing degenerative changes in immobilized discs for more than two years. However, in patients treated with the proposed method, two advantages can be emphasized: (1) the discs are positioned horizontally in the physiological load bearing position, and (2) they are subjected to loads and micromovements allowed by the limited elasticity of the instrumentation. Both these aspects can contribute to the prevention of degenerative disc changes.

The comparison of preoperative mobility prior to the initial corrective surgery is assumed to be altered due to the occurrence of significant scoliotic deformities, especially in this group, which presented with a high number of lumbar curves. We do not know the relevance of this alteration as compared to healthy subjects. The most important aspect is not to determine how much movement is gained or lost, but rather how the spine moves after implant removal. It may be that the range of motion of the spine is not normal after implant removal but exists, contrary to fused spines. This remaining spinal movement will help prevent complications from degeneration of the adjacent segment to the fused area, which is commonly observed in patients with AIS fusion. This complication was prevented by our technique, as shown by the motion of the lower spine segments after implant removal.

Regarding spine regeneration and healthy alignment, we agree with the authors of the letter only on point number two. Our goal in the treatment of adolescent idiopathic scoliosis is to achieve complete correction of vertebral deformity in all three planes (please see tables). We do not agree with the first point because correction in the three planes can be achieved in one surgery alone using our method, while restoration of the vertebral morphology occurs gradually in our cases after curve correction. Complete correction of scoliosis is necessary because respiratory restriction caused by scoliosis can have life-threatening implications. Our observation of permanent respiratory restriction in patients with spinal fusion [3,4], the existence of a respiratory mechanism dependent on the vertebral column in situations of maximum respiratory demands [5], and the demonstration that this respiratory system is abolished in scoliotic children with more than 30° Cobb [6] highlights the importance of preserving vertebral mobility in these patients.

Current treatments for adolescent idiopathic scoliosis with irreversible extensive fusions are unsatisfactory. The technique we describe improves the functional aspects of these patients as well as their quality of life. The rigid instrumentation used in our patients allowed for complete correction of the deformity and guided vertebral growth. The use of more elastic instrumentation would likely make it difficult to achieve proper correction of scoliosis.

## Data Availability

All the data generated or analyzed during this study are included in this published article.
